# Apathy Antedating and Evolving With Dementia: A Case Report and Insights Into Apathy as a Network Dysfunction

**DOI:** 10.7759/cureus.13802

**Published:** 2021-03-10

**Authors:** Hassan Kesserwani

**Affiliations:** 1 Neurology, Flowers Medical Group, Dothan, USA

**Keywords:** alzheimer’s dementia, behavior change

## Abstract

Apathy is a motivational disorder characterized by a lack of drive or indifference to salient endogenous or exogenous influences or stimuli. In the classical literature, it was referred to as a lack of mental energy or vitality and attributed to dysfunction of the ergotropic sympathetic pathway involving the posterior hypothalamus, thalamus, cingulate cortex, and hippocampal formation. Recent advanced imaging studies have expanded the ambit of network dysfunction to include the dopaminergic mesolimbic system and the salience network that gauges the valence or energy of the mesolimbic system. The classic Papez circuit also has a role in the pathogenesis of apathy. In order to understand the mechanisms of apathy seen with a wide range of diseases including neurodegenerative disorders, trauma, and strokes, one needs to think of apathy as a network disorder. In this report, we present a case of apathy antedating and evolving with Alzheimer’s disease and delve into the network theory of apathy and explore potential pharmacological therapies.

## Introduction

Apathy is derived from the Greek word "pathos", which refers to emotion, suffering, or sympathy; apathy, therefore, means lack of emotion. It is a prominent symptom of mood disorders, psychotic disorders, neurodegenerative disorders, strokes, and traumatic brain injuries. Its core clinical features include lack of drive or intention for goal-directed behavior, indifference (neutral interest), and diminished emotional responsiveness (flat affect or emotional blunting) [1]. Paraphrasing in the vernacular, patients with apathy seem to be "vacuous" in both drive and emotion. Frontotemporal dementia (FTD) and progressive supranuclear palsy (PSP) are associated with an exceptionally high prevalence of apathy, with some studies showing the presence of apathy to be as high as 90% in FTD [2]. In comparison, the prevalence of apathy in Alzheimer's disease is close to 50% [3]. Apathy being common to both depression and dementia, the critical distinction between the apathy of depression and the apathy of dementia is that in the former, there is a frank lack of quantitative and qualitative pleasure in activities (anhedonia or sadness). In the latter, the valence or measure of the emotion is neutral: neither increased nor decreased.

The terms abulia, apathy, and akinetic mutism fall on the same clinical trajectory, with apathy synonymous with mild abulia and akinetic mutism synonymous with severe abulia. Abulia is derived from the Greek language, meaning "no will", with the term being associated with lack of willpower more than lack of emotions. An apt term is "psychic akinesia", referring to abulia as an exclusively mental state and not a motor phenomenon, with motor akinesia being an occasional secondary manifestation. Apathy is also referred to as abulia minor, as even though there is diminished will or drive, there is still a limited verbal and motor response. On the other end of the spectrum, there is abulia major or akinetic mutism where there is virtually no motor or verbal response; this is usually due to severe bilateral involvement of the cingulate gyri or connecting pathways, as explained in the Discussion section. On the other hand, anhedonia is usually a core feature of depressive disorders, psychotic disorders, or personality disorders, where there is a frank inability in experiencing or engaging in pleasurable activities [4]. 

Apathy can also antedate dementia and, in this context, may be referred to as bradyphrenia or slowness of thought, a non-specific term that is used to refer to a condition seen in a wide range of disorders including dementia, schizophrenia, or hypokinetic movement disorders, such as Parkinson's disease (PD). In a meta-analysis of 16 studies involving 7,365 patients with subjective memory complaints and mild cognitive impairment, the risk of development of dementia was found to be doubled in those patients with apathy after a follow-up period that ranged from 1.2 to 5.4 years [5].

Apathy is associated with network dysfunction involving the medial frontal cortex, medial orbitofrontal cortex, cingulate cortex, and ventral striatum (VS) [6]. The inferior parietal cortex has also been implicated [7]. In the Discussion section, we will outline the neural network involved in the generation of apathy. At the outset, it is important to note that a lesion in the mesolimbic system may alter the perception of pleasure or the motivation to act. However, if this pathway is intact, then the value of this signal, its valence, maybe under-appreciated by the salience network. This mismatch may lead to apathy. The Papez circuit links the hippocampus with the cingulate cortex, and degeneration of the entorhinal cortex by transsynaptic degeneration may involve the cingulate cortex, leading to apathy [8].

## Case presentation

We present the case of a retired 75-year-old businessman who started experiencing mild memory loss pertaining to appointments, conversations, and close family friend’s names. His pre-morbid personality was dynamic with a happy, cheerful, and engaging temperament, and he was widely versed in domestic and foreign affairs. Over the course of two to three years, he became more introverted and showed no interest in calling family, friends, or socializing. He was content sitting at home and watching television. When questioned about events in his life, answers tended to be neutral and devoid of any information. Conversations were never initiated and he provided simple, binary yes and no answers. Nevertheless, he seemed jovial, unconcerned, and there was no report of sadness, hallucinations, delusions, or agitation. Sleep and nutrition were adequate with a mild decline in hygiene, such as maintaining his nails and bathroom cleanliness after urination. Family gatherings with his grandchildren were not met with the usual joy and happiness; instead, he remained quiet and just kept smiling. His wife stated that he did not appear to be there and seemed to be disengaged from events around him.

His past medical history was significant for coronary artery disease treated with atorvastatin 40 mg daily, hypertension treated with lisinopril 10 mg daily, and atrial fibrillation treated with rivaroxaban 20 mg daily.

On examination, his disposition was neutral and calm, but he did not start any conversation or ask any questions during the interview. Good eye contact was maintained. There was no obvious aphasia; repetition, naming, and reading aloud were normal. Speech prosody and cadence were normal. With immediate attention, he registered three out of seven digits. Delayed recall of three objects at two minutes was zero. There was visuomotor apraxia with geometric hand pantomime and inability to intersect pentagons. Verbal fluency (animal naming) was eight at one minute, with categorical restriction to mammals; no reptiles or other vertebrates, insects, or other invertebrates were mentioned. His mini-mental status score was 19 out of 30. He had difficulty following multiple-step commands. His knowledge of current affairs was scant, but he was able to recollect significant past events such as his wedding and his first business venture.

No frontal lobe signs were noted. Specifically, a snout reflex, jaw jerk, palmomental reflex, hand-grasp reflexes, and paratonia of the arms were all absent. No perseveration of behavior was noted. Limb-kinetic praxis tested with coin deftness and ideomotor praxis tested with transitive actions such as unlocking a door were both normal. Otherwise, gait, cranial nerve, power, and sensory examination were unrevealing.

**Figure 1:**
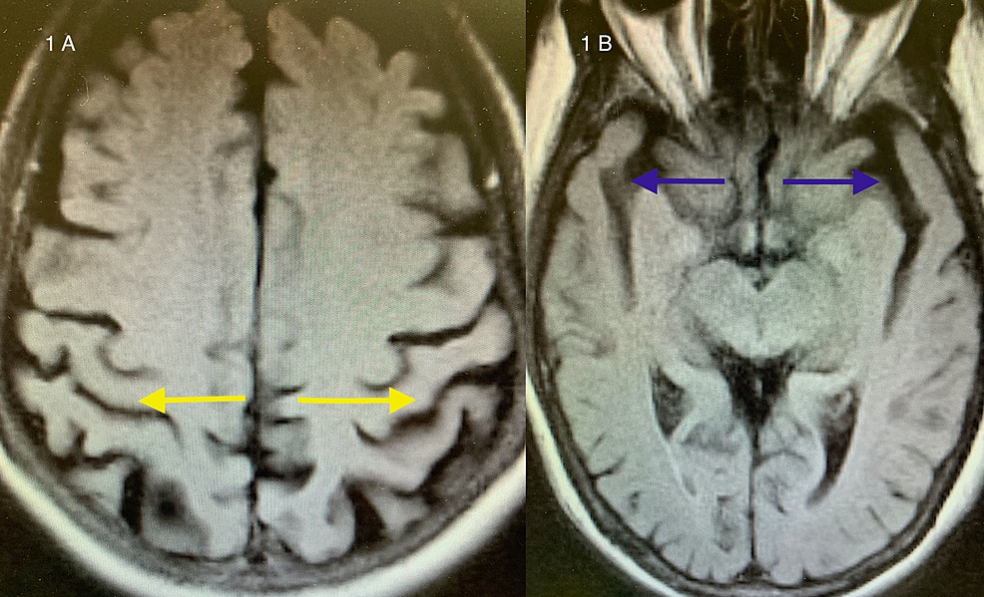
Axial FLAIR MRI of the brain 1A: biparietal lobe atrophy (yellow arrows); 1B: bitemporal lobe atrophy (blue arrows) FLAIR: fluid-attenuated inversion recovery; MRI: magnetic resonance imaging

An MRI of the brain showed bilateral parietal and temporal lobe atrophy typical of that seen with Alzheimer's disease (Figure 1).

A positron emission tomography (PET) scan with an amyloid tag was not deemed necessary, as it would not have altered the management. A bilateral carotid duplex scan was normal. A thyroid screen and serum vitamin B12 were within normal limits.

Based on his gradual cognitive decline, coupled with prominent episodic memory loss and findings on the MRI of the brain, a provisional diagnosis of mild-to-moderate Alzheimer’s disease was made. His lack of motivation and indifference to emotional signals around him, in the absence of depression, led to a diagnosis of apathy. The patient was started on a standard dose of donepezil 5 mg daily and titrated to 10 mg daily at one month. Sadly, his condition continued to deteriorate despite the addition of memantine, and he died of a myocardial infarction five years after the initial diagnosis.

## Discussion

Studies utilizing fluorodeoxyglucose positron emission tomography (FDG-PET) have shown that across a wide spectrum of neurodegenerative disorders including FTD, PD, and PSP, the sine-qua-non of apathy is hypometabolism of the dorsal anterior cingulate cortex (dACC) and the VS (nucleus accumbens and olfactory tubercle) in the basal forebrain. Secondary cortical areas of hypometabolism include the dorsolateral prefrontal cortex (DLPFC) and orbitofrontal cortex (OFC), areas connected with the dACC and VS. These areas along with the fronto-insular cortex are part of the salience network that weighs the emotional content of a stimulus or signal, the so-called valence or emotional value of an incoming message. Other sub-cortical areas with hypometabolism include the thalamus and ventral tegmental area (VTA) (part of the dopaminergic motivation and pleasure pathway) [6].

The VS consists of large cholinergic cells in the basal forebrain that project to most of the neocortex. However, it is the gamma-aminobutyric acid (GABA)-containing medium spiny neurons that are heavily innervated by the dopaminergic mesolimbic system (VTA of the midbrain) via the medial forebrain bundle. This axis represents the classical reward system, integrating motivation and pleasure [9]. Meanwhile, the dACC is connected with the DLPFC and the parietal cortex and is involved in attentional mechanisms. Here reside the von Economo neurons, which are highly specialized large spindle cells with the highest densities in hominids (human and apes). Bilateral ischemic strokes of the dACC can lead to akinetic mutism, and a loss of the von Economo neurons is seen in FTD [10].

In summary, the mesolimbic system (VTA and VS) carries the content of a pleasurable feeling or stimulus via a dopaminergic pathway, which is gauged, weighed, and given value or valence by the dACC, a node of the salience network. A lesion along this cascade can blunt the emotional content or value of the incoming signal and suppress the goal-directed motivation or drive [11].

**Figure 2:**
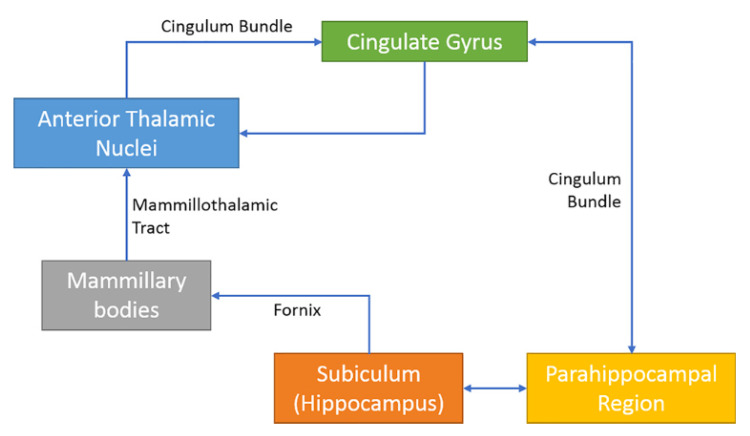
Diagram illustrating the role of the cingulum bundle and cingulate cortex in the emotional Papez circuit Note that the cingulum is part of the conduit from the hippocampus to the cingulate cortex

The role of the cingulate cortex makes intuitive sense as it is a key node in the "emotional" Papez circuit. The cingulum is a major white-matter tract that forms a major limb of the Papez circuit; emerging from the anterior thalamic nucleus, it arches anteriorly above the genu of the corpus callosum all the way posteriorly into the parietal lobe before arching forwards into the parahippocampal gyrus (Figure 2) [12].

In 51 patients with probable Alzheimer’s disease who had apathy, diffusion tensor imaging revealed reduced fractional anisotropy (meaning less dense fiber tracts and hence disconnection) in the left cingulum independent of depression and the use of psychotropic medications. This further cements the role of the cingulate cortex in apathy [13].

The temporal lobe and thalamus in Figure 2 are included to emphasize the role of episodic memory in cognitive function and emotional content. Episodic memory is experiential memory that involves mental time travel and is context-dependent. The core limbic circuit is heavily interconnected with other cortical and subcortical regions and may function more as a memory than an emotional network. However, it is a cohesive network as theta oscillations do resonate through this circuit; these nodes usually degenerate together in various disease states and the hippocampal-cingulate-diencephalic network all contain head-direction cells [14]. Alzheimer's disease is associated with the early loss of neural cells in the subiculum (entorhinal cortex); the latter is connected to the cingulate cortex via the fornix, mammillothalamic tract, and cingulum, putatively explaining the early onset of apathy in Alzheimer's disease patients [15].

The pathway from the posterior hypothalamus and thalamus to the cingulate cortex via the cingulum to the hippocampal formation was formerly known as the ergotropic pathway, ergo referring to sympathetic activation (mental energy). Sectioning of the cingulum in animals led to placidity (calming effect) and to apathy (indifference of events) [16].

Pharmacologically, since the mesolimbic system is dopaminergically driven, there is merit in the use of dopaminergic agonists. The dopamine D3 receptor is rich in the basal forebrain (nucleus accumbens) and pramipexole, which acts through the D2 receptor and may ameliorate motor symptoms and through D3 receptor agonism and may help apathy [17]. Meanwhile, the cholinesterase inhibitor rivastigmine has been shown to be beneficial in a double-blind placebo-controlled study of 31 patients with PD who had apathy but were dementia-free [18].

Only two open-label studies out of eight showed improvement in apathy scores, assessed as part of the Neuropsychiatric Inventory (NPI) or the Clinical Global Impression of Change (CGIC) scales, in patients with mild-to-moderate Alzheimer's disease treated with the cholinesterase inhibitors donepezil and rivastigmine. Two small trials (one randomized, the other open-label) showed improvement with the stimulant methylphenidate at a dose of 10 mg bid with side effects limiting the utility of this drug in patients with Alzheimer's disease. One large randomized controlled trial involving Ginkgo biloba extract also showed statistically significant improvement in apathy scores. The addition of modafinil to donepezil in one study of 23 patients with Alzheimer's disease showed no improvement of apathy [19].

There is a need for randomized placebo-controlled trials about the treatment of apathy, preferably studies utilizing PET imaging of the brain to determine the network involved in the generation of apathy as this may help determine the class of drugs one may use. If the mesolimbic system is involved, one may consider a dopaminergic agent as opposed to a cholinesterase inhibitor if the basal forebrain and hippocampus are involved. The white-matter tracts of these networks may be further analyzed with diffuse tensor imaging, using the connectome library.

## Conclusions

Apathy used to be a seemingly nebulous and non-specific concept. However, with the advent of advanced imaging technology, the understanding of apathy has expanded from a lack of mental energy to a network dysfunction concept pertaining to a lack of mesolimbic innervation of the basal forebrain and abnormal calibration of the valence of the mesolimbic pathway by the salience network. The core dysfunction of the cingulate cortex in a wide variety of diseases associated with apathy concurs with prior clinicopathological studies and the classical circuit of Papez. In this article, we argue that the involvement of the entorhinal cortex early in Alzheimer’s disease, by downstream effects via the fornix, mammillothalamic tract, and cingulum to the cingulate cortex, may explain the presence of apathy in affected patients. Future connectome studies with diffusion tensor imaging may clarify the picture further. Another area in need of further research is the pharmacotherapy or neuromodulation of apathy.
